# Cytoplasmic Tail of MT1-MMP: A Hub of MT1-MMP Regulation and Function

**DOI:** 10.3390/ijms24065068

**Published:** 2023-03-07

**Authors:** Katerina Strouhalova, Ondřej Tolde, Daniel Rosel, Jan Brábek

**Affiliations:** 1 Department of Cell Biology, Charles University, Viničná 7, 128 00 Prague, Czech Republic; 2 Biotechnology and Biomedicine Centre of the Academy of Sciences and Charles University (BIOCEV), Průmyslová 595, 252 50 Vestec u Prahy, Czech Republic

**Keywords:** MT1-MMP, matrix metalloproteinases, cell invasion, intracellular trafficking, post-translational modifications

## Abstract

MT1-MMP (MMP-14) is a multifunctional protease that regulates ECM degradation, activation of other proteases, and a variety of cellular processes, including migration and viability in physiological and pathological contexts. Both the localization and signal transduction capabilities of MT1-MMP are dependent on its cytoplasmic domain that constitutes the final 20 C-terminal amino acids, while the rest of the protease is extracellular. In this review, we summarize the ways in which the cytoplasmic tail is involved in regulating and enacting the functions of MT1-MMP. We also provide an overview of known interactors of the MT1-MMP cytoplasmic tail and the functional significance of these interactions, as well as further insight into the mechanisms of cellular adhesion and invasion that are regulated by the cytoplasmic tail.

## 1. Introduction

Cell invasion is a process cells utilize in a wide range of situations, such as cancer cell metastasis, angiogenesis, macrophage motility, or during development [1]. MT1-MMP (Membrane Type 1 Matrix Metalloproteinase) is essential for a mesenchymal mode of invasion. It is one of the main components of podosomes and invadopodia, extracellular matrix (ECM) contacts which, thanks to MT1-MMP and other matrix metalloproteinases, have the capacity to degrade ECM components [1].

Initially, invadopodia were described as ECM-degrading actin-rich puncta in 2D experiments where cells were seeded typically on gelatine-coated glass cover slips (for reviews, see [1,2]). A detailed description of the structure of invadopodia in a 3D environment was initially resolved by electron microscopy using a thick Matrigel layer [3]. In this environment, invadopodia exhibit a pseudopodia-like appearance [3]. Using a dense fibrillar 3D collagen (such as a skin-based matrix or high-density fibrillar collagen) usually leads to a formation of invadopodia with a protruding base from which numerous thin filopodia-like filaments extend [4,5]. This phenotype was recently confirmed using a detailed 3D CLEM (correlative light-electron microscopy) visualization combining confocal and FIB-SEM (focused iron beam scanning electron microscopy) imaging [6]. Although the initial structure can form in the absence of MT1-MMP, invadopodia elongation into the matrix is dependent on the presence of MT1-MMP, as is their degradative function [7,8].

MT1-MMP is a member of matrix metalloproteinases (MMPs), zinc-dependent enzymes which degrade ECM components. MMPs can be separated into two subgroups–soluble and membrane-type MMPs. The membrane type subgroup comprises MMPs that either contain a transmembrane domain (MT1-, MT2-, MT3-, MT5-MMP) or a glycosylphosphatidylinositol (GPI) anchor, which tethers them to the surface of the cell (MT4-, MT6-MMP) [9].

MT1-MMP is synthesized as a proMT1-MMP zymogen with an inhibitory prodomain, a catalytic domain (CAT), a hemopexin domain (HPX), which serves for dimerization, substrate binding, and phospholipid bilayer interactions [10], a trans-membrane domain (TM), and a short cytoplasmic tail (CT) [11,12]. As a protease, MT1-MMP has an ample portfolio of substrates, including many ECM components, other MMPs, receptors, and other proteins, and it also serves as a signaling hub (for reviews, see [13,14]. It is expressed in invasive cancer cells and many other cell types, such as fibroblasts, endothelial cells, and cells of the immune system [15].

**Figure 1:**
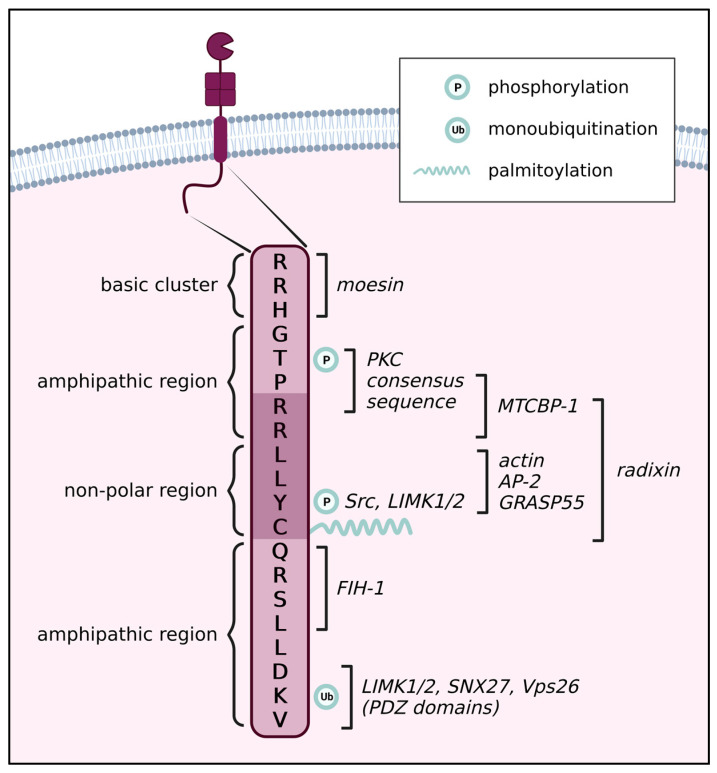
Overview of the cytoplasmic tail of MT1-MMP and its main interactors. The cytoplasmic tail (CT) contains one basic cluster, one non-polar region, and two amphipathic regions. Residues Arg^569^-Cys^574^ can form a β-sheet-like structure (dark purple background). Thr^567^ and Tyr^573^ can be phosphorylated, Lys^581^ can be ubiquitinated, and Cys^574^ can undergo palmitoylation. Interacting partners and known binding sites are depicted on the right side of the diagram.

The cytoplasmic tail comprises the last 20 C-terminal amino acids of the protein, residues 563–582 (human sequence numbering used throughout, UniProt ID P50281). It consists of four regions: a juxtamembrane basic cluster (^563^RRH^565^), an amphipathic region (^566^GTPRR^570^), a nonpolar region (^571^LLYC^574^), and a final amphipathic region (^575^QRSLLDKV^582^). The central part of the CT (^569^RRLLYC^574^) can form a β-strand structure, which contains a β-bulge due to the insertion of an extra leucine (Leu^571^) [16] (Figure 1). Despite its meager length, the CT contains multiple amino acids, which are post-translationally modified, and several binding sites for intracellular proteins, as summarized in Figure 1. Thanks to these interactions, this short tail serves as a hub of incoming and outgoing signaling. In this review, we provide an overview of how MT1-MMP and its functions are regulated through the CT and, in return, how it uses the CT to modulate signaling in the cell.

## 2. Post-Translational Modifications

Following its synthesis, the activity, localization, and function of MT1-MMP are determined by a plethora of modifications. We will discuss the contribution of the cytoplasmic tail to the glycosylation of the enzyme, activation of the zymogen, and self-proteolysis, and the sites of phosphorylation, palmitoylation, and ubiquitination that have been identified within it. In this chapter, we will describe the modifications as such, and they will be revisited in a later chapter in regard to their effect on MT1-MMP trafficking or function.

### 2.1. The Role of the CT on the Regulation of O-Linked Glycosylation

MT1-MMP performs most of its functions on the cell surface. Therefore, such as most other membrane proteins, it is glycosylated. According to software predictions and experimental evidence, it seems that MT1-MMP is not *N*-glycosylated [17,18]. The hinge region between the CAT and HPX domains is subject to *O*-linked glycosylation, specifically on four residues: Thr^291^, Thr^299^, Thr^300^, and Ser^301^. Glycosylation seems to regulate only a certain subset of MT1-MMP functions, given that collagen proteolysis and autoprocessing are unaffected, but it is essential for MMP2 activation [17,18].

The mutation of the cytoplasmic dileucine motif LL^572^ was shown to impact the glycosylation pattern of MT1-MMP. The following Tyr^573^ was also assessed for a potential role in glycosylation regulation, as it often acts as a unit with LL^572^, but it does not seem to have a pronounced effect here. The role of LL^572^ in ensuring proper glycosylation of MT1-MMP seems to be unrelated to the well-studied role of LLY^573^ in MT1-MMP trafficking (see Section 4), as glycosylation occurs in Golgi prior to prodomain cleavage [18].

The mechanism underlying the function of LL^572^ in glycosylation remains unknown, but MT1-MMP was found to bind GRASP55 (Golgi reassembly-stacking protein of 55 kDa) via the LLY^573^ motif [19]. GRASP55 is responsible for the morphology of the Golgi apparatus and certain unconventional modes of secretion, but it has also been found to regulate the glycosylation of proteins and also spatially organize glycosylation enzymes within the Golgi [20,21,22]. It is possible that the interaction with GRASP55 or a similar adaptor protein facilitates proper glycosylation of MT1-MMP.

### 2.2. The Role of the CT in MT1-MMP Activation via Prodomain Cleavage

MT1-MMP is synthesized as a zymogen—a latent form of the enzyme where the catalytic site is blocked by a prodomain. The initial form of MT1-MMP activation is the removal of the prodomain by furin or other proprotein convertases (PCs) either in the Golgi or after secretion [23,24]. However, the soluble prodomain is also capable of inhibiting MT1-MMP and must be cleaved by MT1-MMP itself to be degraded [25].

Interestingly, although the CT domain is positioned on the other side of the protein and is located on the inside of the cell, unlike the extracellular prodomain, it regulates the processing of the prodomain. The deletion of the CT leads to a strong reduction in prodomain cleavage [26]. As mentioned above, the LLY^573^ motif in the CT binds GRASP55, which also interacts with furin and could therefore act as a potential adaptor for these two proteins during MT1-MMP activation. The disruption of this interaction did lead to a reduction in MT1-MMP activation, albeit a small one [19]. On top of that, the substitution of the CT for the CT of MT2-MMP, which is not identical but does contain the LLY motif and was found to also associate with GRASP55 [19], also partially blocked MT1-MMP prodomain cleavage [26]. It seems that the interaction with GRASP55 is not the only one that assures proper activation of the zymogen. This could be due to activation by other PCs, which may not bind GRASP55 and use a different mechanism to access MT1-MMP or the presence of multiple adaptors.

### 2.3. The Role of the CT in MT1-MMP Autoprocessing

One form of regulating MT1-MMP activity is the removal of the catalytic domain, which results in the production of a species composed of the HPX, TM, and CT domains. The actual molecular weight of this degradation product is 37.7 kDa, but it is usually referred to as a 43 or 44 kDa form due to how it migrates on SDS-PAGE [27]. The formation of this species is an autocatalytic process facilitated by a second molecule of MT1-MMP. It requires dimerization of the “substrate” and “catalytic” molecules of MT1-MMP and is associated with MMP2 activation [27,28,29,30].

The cytoplasmic tail has been shown to play a role in MT1-MMP autoprocessing [31]. Apart from Cys^574^, whose involvement in homodimerization will be discussed later (Section 3), the RRH^563^ motif, which is the binding site of moesin, an ERM (ezrin, radixin, moesin) protein, seems to be particularly important. ERM proteins link transmembrane proteins to the actin cytoskeleton and act as organizers of plasma membrane domains [32]. The RRH^563^ motif is necessary for MT1-MMP clustering in tetraspanin-enriched domains in cell protrusions. This clustering, which likely mediates the trans-autocatalytic removal of the CAT domain by bringing MT1-MMP spatially closer together and thus allowing dimerization, is proposed to occur thanks to the interaction with moesin [33].

### 2.4. Palmitoylation of the CT

MT1-MMP was identified as a substrate of Zinc Finger DHHC-Type Palmitoyltransferase 13 (ZDHHC13) [34]. In particular, the cytoplasmic cysteine at position 574 is palmitoylated [35]. This modification causes the tethering of the CT to the plasma membrane (PM). Therefore, the proposed model is that it positions neighboring residues in an optimal way to allow for membrane-bound proteins to interact with them [35]. Mutation of Cys^574^ to alanine or serine results in aberrant localization of MT1-MMP in multiple cell types [34,35]. Palmitoylation at Cys^574^ affects clathrin endocytosis and cell motility (further discussed later).

### 2.5. Phosphorylation of the CT

MT1-MMP contains three potential phosphorylation sites in its cytoplasmic tail, Thr^567^, Tyr^573^, and Ser^577^. The former two have been confirmed to be phosphorylated in cells, and the effect of their phosphorylation on adhesion, migration, invasion and other processes has been extensively studied. Here we provide an overview of the mechanisms of phosphorylation, while the specific roles of Thr^567^ and Tyr^573^ phosphorylation in MT1-MMP regulation and MT1-MMP-mediated cellular processes will be discussed in respective chapters.

#### 2.5.1. Thr^567^

Thr^567^ was shown to be phosphorylated by the serine/threonine kinase Protein Kinase C (PKC) in vitro [36] and in response to PKC activation by phorbol 12-myristate 13-acetate (PMA) treatment in cancer cells [37], but not in a purely physiological context. The silencing of another PKC superfamily member, atypical PKC iota (aPKCι), led to the disruption of MT1-MMP trafficking. It also colocalized with MT1-MMP at cell-cell contacts and in vesicles, but whether MT1-MMP is a substrate of aPKCι has not been determined [38]. Thr^567^ phosphorylation was also observed in response to β1 integrin activation, which occurs upon adhesion to the ECM. Activation of β1 integrin causes the recruitment of the Src kinase and subsequent Epidermal Growth Factor Receptor (EGFR) phosphorylation upstream of Thr^567^ phosphorylation [39,40].

PKC interacts with tetraspanins, transmembrane proteins that act as scaffolds for organizing membrane domains, which link it to β1 integrin [41,42,43]. Simultaneously, MT1-MMP is also known to bind many tetraspanins [44]. It has been proposed that tetraspanins facilitate MT1-MMP proteolytic function by bringing MT1-MMP and its substrates together [45]. It is then feasible that tetraspanin-enriched domains also cluster MT1-MMP, PKC, and β1 integrin together to allow PKC-mediated Thr^567^ phosphorylation in response to β1 integrin activation.

#### 2.5.2. Tyr^573^

Despite not being embedded in any canonical tyrosine kinase recognition motif, Tyr^573^ was found to be phosphorylated in COS-7 cells overexpressing MT1-MMP and the tyrosine kinase Src and in response to EGF treatment, which activates Src, in OVCA433 cells overexpressing MT1-MMP [46,47]. Expression of a dysfunctional Src mutant leads to the loss of Tyr^573^ phosphorylation, confirming that either Src itself or one of its downstream effector kinases phosphorylates MT1-MMP at this residue [46]. Tyr^573^ was also found to be phosphorylated by LIMK1/2 (LIM domain containing kinase 1 and 2), which binds MT1-MMP via DKV^582^ at the very C terminus of the CT [48]. In endothelial cells, Tyr^573^ phosphorylation increases upon stimulation with sphingosine-1-phosphate, a signaling molecule naturally abundant in blood [46,49].

### 2.6. Ubiquitination of the CT

Ubiquitination is mostly known to target proteins for degradation; however, it also regulates many non-proteolytic cellular processes, such as protein-protein interactions, protein activity, or localization [50]. In the case of MT1-MMP, monoubiquitination of Lys^581^ by the E3 ubiquitin-protein ligase NEDD4 (neural precursor cell expressed developmentally down-regulated protein 4) regulates its trafficking and, therefore, function. A prerequisite for Lys^581^ monoubiquitination is the phosphorylation of Tyr^573^ [51].

## 3. The Role of the CT in Homodimerization

MT1-MMP multimerization is necessary for cellular processes such as adhesion, collagenolytic, and 3D matrix invasion [52,53,54], as well as for MT1-MMP self-regulation in the form of autoprocessing [31]. All four domains of the active proteinase—CAT, HPX, TM, and CT—have been implicated in facilitating homodimerization [31,55,56,57,58]. However, the contribution of the cytoplasmic tail has been a subject of debate. Studies have shown conflicting results, with some providing evidence that CT is, in fact, essential for dimerization [31,52], while others argue it is not [35,56].

It was proposed that the observation of dimers in some experiments was a result of artefactual disulfide bridge formation in lysates [35]. Nonetheless, experiments that use iodoacetamide, an inhibitor that covalently binds free cysteine thiol groups and therefore prevents disulfide bridge formation in lysates, confirmed the necessity for an intact CT, Cys^574^ in particular, during multimerization. They also showed an identical multimerization pattern in regular lysates and lysates where iodoacetamide was added to the lysis buffer [52].

Furthermore, expression of a truncated construct containing the TM and CT domains of MT1-MMP was sufficient to block autoprocessing to the 43 kDa form, which occurs in *trans* and requires homodimerization [30], likely through the TM-CT construct acting as a competitive partner for dimer formation [31]. Itoh et al. have shown that the TM itself is sufficient for dimer formation. Therefore, it is unclear which domain was responsible [56].

Another process for which homodimerization of MT1-MMP is needed is the activation of MMP2. This activation occurs in a complex consisting of an MT1-MMP dimer, where one of the molecules acts as a tether for TIMP2 (tissue inhibitor of metalloproteinases 2) bound to proMMP2, bringing the proenzyme into the proximity of the other MT1-MMP molecule, which carries out the enzymatic reaction [15]. Interestingly, tempering with the CT did not greatly affect the activation of MMP2, regardless of whether the experiments showed that the CT contributed to multimerization or not [52,56,59].

As discussed earlier, Cys^574^ is palmitoylated, which leads to the tethering of the CT to the membrane. Thus, Cys^574^ might contribute to homodimerization by maintaining MT1-MMP clustered in certain regions of the plasma membrane, bringing the monomers to close together.

## 4. The Influence of the CT on MT1-MMP Trafficking and Localization

**Figure 2:**
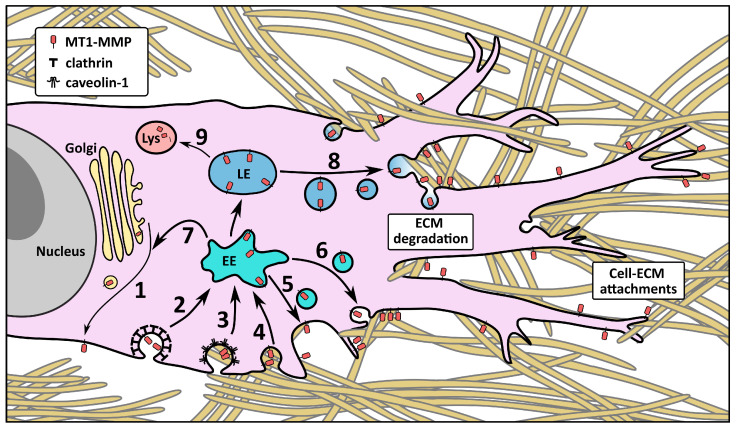
Overview of the main trafficking pathways of MT1-MMP. MT1-MMP is subjected to extensive trafficking through the cell as a form of regulation. The main sites of degradation are where the cell body is constricted by ECM fibers in the direction of migration [64]. (1) Biosynthetic Rab8-dependent pathway. (2) Endocytosis in clathrin-coated pits. (3) Endocytosis in caveolae. (4) Endocytosis in flotillin-rich microdomains. (5) Fast Rab4/Rab14-dependent recycling. (6) Slow Rab11-dependent recycling. (7) Recycling through the Rab8-positive compartments. (8) Recycling from Rab7-positive LEs to sites of degradation. (9) Progression to the lysosome for degradation. EE—early endosome; LE—late endosome; Lys—lysosome.

Compartmentalization is a significant mechanism of regulation of MT1-MMP. It undergoes complex context-dependent trafficking through the cell and into and out of distinct PM domains (summarized in Figure 2). It has been observed in specialized domains–tetraspanins, flotillins, caveolae [29,44,60,61,62], in invadopodia [63], as well as in early, late, and several types of recycling intracellular compartments. Many of these events are orchestrated by interactions of various proteins with the CT.

### 4.1. Membrane Localization and Endocytosis

The CT is the main region that facilitates the internalization and movements of MT1-MMP through the cell. Its deletion markedly decreases the uptake of MT1-MMP into cells and increases its surface localization [59,61,65,66]. Three pathways of MT1-MMP endocytosis have been observed–clathrin-, caveolae-, and flotillin-dependent.

In clathrin-mediated endocytosis, molecules of the coat protein clathrin are recruited to the PM. Their accumulation causes a curving of the membrane, resulting in the formation of pits. Further recruitment of clathrin deepens the pits into vesicles, which are then pinched off into the cytosol [67]. Studies showed that MT1-MMP is taken up in clathrin-coated pits (CCPs) into Early endosome antigen 1 (EEA1)-positive early endosomes (EEs), an event which was blocked by the deletion of the CT [65]. The internalization rate when the CT is removed is slower (none was observed within the first 5 min, and it was reduced by about 60% at the 30-min mark compared to wild-type (WT)) and occurs in a caveolae-dependent manner [35,59], indicating that the CT in indispensable for clathrin-mediated endocytosis. Endocytosis in CCPs is mediated by the interaction of the LLY^573^ motif in the MT1-MMP CT with the µ2 subunit of AP-2, a CCP cargo adaptor protein [59]. The palmitoylation of the following Cys^574^ is also critical, likely facilitating this interaction by bringing the LLY^573^ motif close to the membrane [35].

There have been many studies confirming and disproving the physiological relevance of caveolae-dependent endocytosis of MT1-MMP. Caveolae lipid rafts are membrane domains that form PM invaginations and are involved in clathrin-independent endocytosis, lipid homeostasis, and cell signaling. They are rich in cholesterol, sphingolipids, and their resident protein, caveolin-1 [68,69]. A couple of reports observed very little presence of endogenous WT MT1-MMP in caveolar membrane fractions, identifying caveolae-dependent endocytosis as a substitute mechanism in the case of CT deletion, and therefore considered it physiologically irrelevant and not dependent on the CT [35,66].

However, other studies obtained contradictory results, finding MT1-MMP in caveolin-1-positive membrane domains and vesicles [29,61,62] and observing partial inhibition of MT1-MMP internalization when caveolae were disrupted in cancer cells [47,61,70]. Yang et al. showed that MT1-MMP colocalized with caveolin-1 at invadopodia of breast cancer cells in response to fluid shear stress, a factor cancer cells encounter when metastasizing through blood and lymphatic vessels [71]. MT1-MMP localization to caveolae seems to be particularly abundant in endothelial cells, where blocking clathrin-dependent endocytosis has almost no effect, whereas disrupting caveolae has a pronounced impact on MT1-MMP internalization [72]. Furthermore, MT1-MMP was found to associate with caveolin-1 upon vascular endothelial growth factor (VEGF) stimulation of endothelial cells. This interaction may not be direct, as both the C- and N-termini of caveolin-1 face the cytosol, and the MT1-MMP CT lacks a consensus caveolin-binding motif [73,74,75]. In any case, it requires Cys^574^ and Val^582^ from the CT [76]. Similarly, epidermal growth factor (EGF) stimulation of ovarian cancer cells leads to the localization of MT1-MMP to caveolae and internalization in caveolin-1-positive vesicles [47].

Flotillin microdomains are another type of lipid raft that is independent of caveolae. They are rich in sphingolipids and cholesterol, facilitate receptor clustering, and, when overexpressed, form invaginations leading to endocytosis [77]. MT1-MMP was shown to be internalized in these microdomains, but the role of the CT in flotillin-dependent endocytosis has not been addressed. However, flotillins are cytosolic, and MT1-MMP was found to coimmunoprecipitate with flotillin-1. Therefore, it is probable this interaction occurs thanks to the CT, be it direct or otherwise [60].

MT1-MMP internalization is also modulated through Thr^567^, whose phosphorylation promotes the process, as was shown in experiments where PMA-induced phosphorylation or the expression of a phosphomimetic lead to higher endocytosis. PMA treatment resulted in co-internalization and co-trafficking with α5β1 integrin [37]. Endocytosis is further dependent on the integrity of the juxtamembrane RRH^563^ region, which, as mentioned in Section 2.3., ensures clustering in tetraspanin-enriched domains [33]. These domains are where β1 integrin is also enriched and linked to PKC, which phosphorylates both β1 integrin and Thr^567^ of MT1-MMP [33,41]. Finally, the ubiquitination of Lys^581^ upon Tyr^573^ phosphorylation downregulates the uptake of MT1-MMP into cells [51].

Regardless of the endocytic mechanism, MT1-MMP is found in vesicles with the early endosome markers Rab5 or EEA1 in many cell types and contexts [37,40,60,61,65,78,79]. Cells regulate endocytosis of MT1-MMP to control the amount of available MT1-MMP on the surface, such as in the case of nutrient starvation, which blocks clathrin endocytosis via mTOR (mammalian target of rapamycin), sequesters MT1-MMP on the surface, thus assuring abundant proteolysis of ECM (e.g., fibronectin or type I collagen) to create a nutrient source for starved cells [80]. Conversely, endocytosis is also a contributor to proper MT1-MMP function, for example, by taking up MT1-MMP inhibited by TIMP2, which is then released from the protease in the acidic pH of endosomes [61,81].

### 4.2. Intracellular Trafficking and Recycling

Endocytosed MT1-MMP can travel through many routes inside the cell, mostly through various modes of recycling. A major pathway, which has been observed in a large number of studies, is the progression from EEs to Rab7, VAMP7 (Vesicle Associated Membrane Protein 7), and LAMP-1 (Lysosomal Associated Protein 1) containing late endosomes (LEs) and on-demand recycling to the PM [37,61,82,83,84,85].

MT1-MMP has also been found in Rab4 or Rab14-positive fast recycling vesicles [61,78,86], Rab11-positive slow recycling vesicles [87], Rab22-positive recycling clathrin-independent endosomes [78], and Rab8-positive compartments, which can be either involved in exocytosis in the biosynthetic pathway or in recycling as well [78,87]. The selection of the recycling process depends on the context and cell type.

Of particular interest is the delivery of MT1-MMP to invadopodia, which has been described in many publications and reviewed in detail previously [63,88]. The translocation of MT1-MMP-positive LEs to the invadopodial PM was shown to be triggered by the association of Srcasm (Src activating and signaling molecule) with TOLLIP (Toll interacting protein), an endosomal sorting protein [82]. Similarly, the ER protein Protrudin was shown to make contacts with MT1-MMP-loaded LEs containing the kinesin-1 adaptor FYCO1 (FYVE And Coiled-Coil Domain Autophagy Adaptor 1), thus assuring LE translocation to the invadopodial PM and exocytosis [8]. The microtubule-associated motor protein kinesin-1 is recruited to MT1-MMP LEs by JIP3/JIP4, which in turn are recruited by the WASH (Wiskott-Aldrich syndrome protein and scar homolog) complex [89]. WASH is also part of a complex that forms when MT1 LEs establish contact with the PM. Apart from WASH, it includes F-actin, cortactin (an invadopodial component that modulates actin polymerization), and the exocyst (a vesicle tethering complex) [90]. The exocyst complex, which ensures MT1-MMP delivery to invadopodia, is activated by RhoA and Cdc42 by way of triggering the interaction of the Sec3 and Sec8 subunits of the complex [91].

Once the vesicle arrives at the PM, membrane fusion is mediated by target membrane (t) and vesicle membrane (v) SNAREs (SNAP [Soluble N-ethylmaleimide-sensitive factor attachment protein] receptors). Two SNARE complexes have been described to specifically facilitate fusion at invadopodia: a complex composed of the tSNAREs SNAP23 and syntaxin 4 and the vSNARE VAMP7 [83,84], and an unconventional complex of Bet1 and its interacting SNAREs Vti1B (vSNARE) and syntaxin 4 (tSNARE) [85]. Additionally, another complex was identified, and although the exact site of the vesicle delivery was not determined in the experiments, it was shown to be crucial for ECM degradation and invasion. It comprises of the vSNARE VAMP3, which directs the exocytosis of microvesicles containing MT1-MMP [92] and MT1-MMP exocytosis from Rab4 recycling vesicles [93], and tSNARES SNAP23 and syntaxin 13 [94].

Taken together, many proteins facilitating the delivery of MT1-MMP to the membrane have been identified, but our understanding of the involvement of the CT in most of the aforementioned pathways is poor. Although we can assume it is important for vesicle cargo selection, which decides the fate of each molecule, and adaptor binding, there is currently little experimental evidence that describes these interactions.

The contribution of the C-terminal DKV^582^ motif has been described. Its disruption arrested MT1-MMP-containing vesicles inside the cell [86], and it was found to interact with sorting nexin 27 (SNX27) and the Vps26 subunit of the retromer, a protein complex responsible for sorting transmembrane proteins from the endosomes [95]. This interaction promotes MT1-MMP recycling, presumably from LEs, because the retromer interacts with the WASH complex and Rab7 and recruits’ cargo in response to sequential Rab5 and Rab7 signaling [96,97,98,99].

## 5. The Role of the CT in Downstream Signalling of MT1-MMP

**Figure 3:**
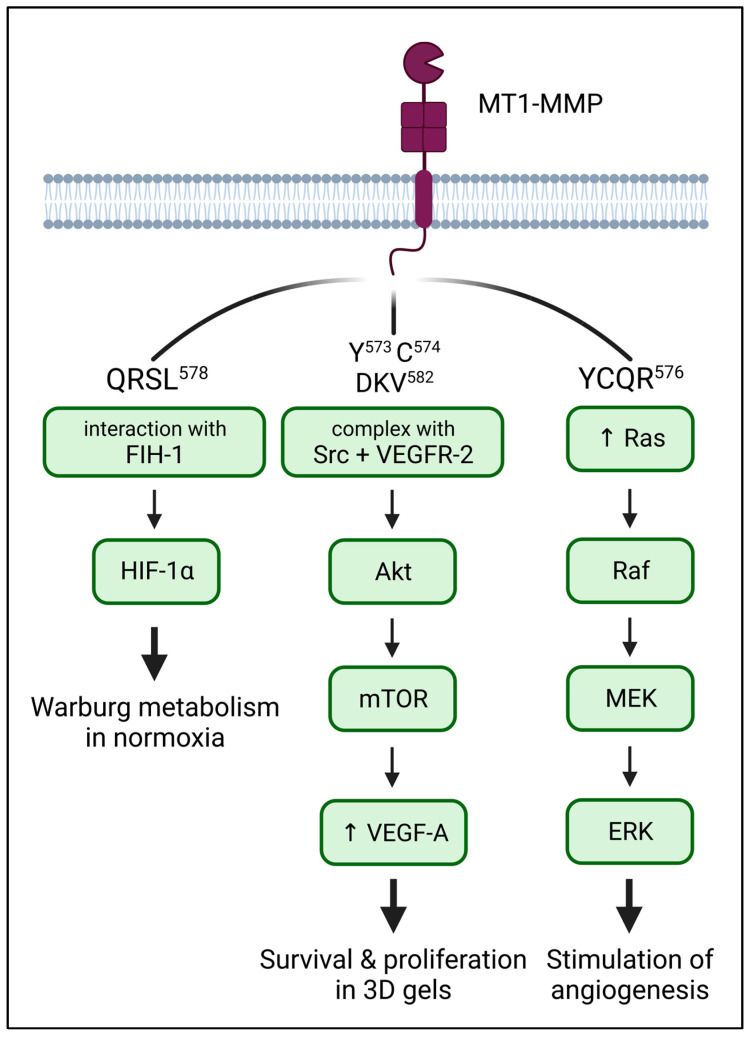
Simplified overview of signaling pathways downstream of MT1-MMP dependent on the CT. The QRSL^578^ motif is responsible for binding FIH-1, an inhibitor of HIF-1α, leading to HIF-1α activation and sustained Warburg metabolism in normoxic conditions (**left**). MT1-MMP forms a complex with Src and VEGFR-2, promoting VEGF-A expression (**center**). Through an unknown mechanism, the YCQR^576^ sequence is responsible for the induction of the ERK pathway in response to adhesion (**right**). All three pathways also induce the expression of MT1-MMP.

The original perception of MT1-MMP solely as an MMP2 activator and ECM degradation enzyme has long been surpassed by the notion of MT1-MMP as a multifaceted player in many cellular processes. MT1-MMP is able to assume a receptor-like role, transducing signals from the exterior of the cell inside via its cytoplasmic tail and modulating signaling therein (Figure 3).

### 5.1. HIF-1α: Metabolism

MT1-MMP was identified as an inducer of Warburg metabolism, a metabolic strategy employed by certain cell types where anaerobic glycolysis is used even in normoxic conditions [100,101]. In macrophages and cancer cells, MT1-MMP activates HIF-1α (subunit α of hypoxia-inducible factor 1) by associating with asparaginyl hydroxylase FIH-1 (factor inhibiting HIF1-α) in normoxic conditions through its CT. Namely, the QRSL^578^ sequence is important for this interaction, especially Arg^576^, whose mutation abolishes FIH-1 binding completely. The interaction with MT1-MMP sequesters HIF-1α in the Golgi, preventing it from binding and inhibiting HIF-1α in the cytosol [102,103]. Additionally, the expression of MT1-MMP in cancer cells which do not normally express it, is enough to induce the Warburg metabolism [103]. MT1-MMP, therefore, seems to be the key to maintaining hypoxic metabolism even in normoxic conditions.

Additionally, in hypoxic conditions, MT1-MMP transcription is induced in a HIF-1α-dependent manner [104,105]. Therefore it is possible that a positive feedback loop is also established when HIF-1α is upregulated in the context of normoxia, thus reinforcing the effect.

### 5.2. ERK: MAPK Signaling

The activation of MAPK (mitogen-activated protein kinase) ERK1/2 (extracellular signal-regulated kinase 1/2) downstream of a Ras-Raf-MEK1/2 (MAPK/ERK kinase 1/2) pathway in response to growth factors is one of the most studied signaling pathways [106,107,108]. However, ERK activation also occurs upon attachment to ECM, which requires MT1-MMP and is dependent on the CT and the presence of TIMP2 [109,110,111,112]. The exact interactor(s) that transduce(s) the message is/are not known, but the YCQR^576^ region in the CT is necessary for it. Mutation of Tyr^573^ or Cys^574^ to alanine activates ERK irrespective of whether TIMP2 is present, while the phosphomimetic Y573D mutation leads to no ERK activation. Tyr^573^ phosphorylation is, therefore, a mechanism of downregulation of the ERK pathway [111]. Expression of MT1-MMP, along with the presence of TIMP2, also upregulated Ras expression and Raf phosphorylation [111]. MT1-MMP-mediated activation of ERK results in survival and proliferation in 3D gels [113]. At the same time, activation of the ERK pathway leads to higher MMP2 activation, which is MT1-MMP dependent, and sustained ERK signaling also increases MT1-MMP levels, indicating a positive feedback loop [70,110,114].

### 5.3. VEGF: Stimulation of Angiogenesis

Overexpression of MT1-MMP in cancer cells leads to an induction of vascular endothelial growth factor (VEGF) expression and stimulation of tumor angiogenesis in vivo [115,116,117]. MT1-MMP upregulates specifically VEGF-A, independently of ERK, p38, or phosphatidylinositol 3-kinase, but inhibition of the Src family of kinases reduces the effect [117]. MT1-MMP was found to form a complex with VEGFR-2 (VEGF receptor 2) and Src, leading to the activation of Akt and mTOR, ultimately stimulating VEGF-A expression [118]. This process requires the CT, primarily Tyr^573^, Cys^574^, and DKV^582^ [34,117,118]. Similarly to the two aforementioned pathways, VEGF also induces MT1-MMP expression, reinforcing the signaling pathway [119].

## 6. The Importance of the CT for Cell Adhesion

MT1-MMP has several functions in cellular adhesion, although the role of CT in this process is less clear. Many studies have demonstrated a functional interaction between MT1-MMP and integrins, its role in the processing of pro-α subunits, and MT1-MMP localization with β1 integrins at distinct cell compartments [13,120,121]. So far, to our knowledge, only one study showed functional interaction between integrins and the CT of MT1-MMP. MT1-MMP-GFP was found colocalizing with β1 or αvβ3 integrins at cell-cell contacts and motility structures, presumably focal adhesions (FAs) and lamellipodia, of migrating endothelial cells on collagen I, while the CT deletion mutant could rarely be observed at those cell sites colocalizing with integrins [122]. β1 integrin association with MT1-MMP might directly interfere with and/or induce modifications around its internalization motif, also affecting the multimerization site (Cys^574^) required for its full function [31,122] (see Section 3). However, it is possible that this effect was a consequence of impaired recycling or due to other signaling events that were discovered later, and the main functional interaction between integrins and MT1-MMP is within their extracellular domains.

CD44 is an important adhesion molecule and a major receptor for hyaluronic acid. However, due to a glycosylation pattern that is also manipulated by alternative splicing, it can establish a number of indirect interactions with other ECM components (collagen, fibronectin, laminin, and several growth factors) [123]. CD44 interacts with MT1-MMP through the HPX domain and directs it to lamellipodia [124]. The interaction through HPX is critical for CD44 shedding by MT1-MMP [125], which causes the cell to detach from the ECM and thus stimulates cell migration [126]. The juxtamembrane region of the CD44 cytoplasmic tail directly binds to the N-terminal FERM domain of radixin [127] and moesin [33] and is thus connected to filamentous actin. It was shown that radixin simultaneously binds the CTs of MT1-MMP and CD44 [16]. MT1-MMP binding to subdomain A of the radixin FERM domain has no overlap with CD44 binding to subdomain C, therefore a stable ternary complex comprising MT1-MMP, ERM proteins, and CD44 could form at the invasive front [16]. Concurrently, ERM proteins bring MT1-MMP and CD44 into close proximity and accelerate CD44 shedding by MT1-MMP. The CT of MT1-MMP thus has a direct role in cell adhesion and detaching through ERM protein-mediated colocalization of MT1-MMP and CD44.

The interaction interphase of MT1-MMP with radixin was located at a six amino acid sequence at the very end of the CT (569–574) that overlaps with the phosphorylation site at Tyr^573^, though it is not clear whether this modification imposes any constrains on the interaction or other functional interferences. The phosphorylation of MT1-MMP at Tyr573 is induced by sphingosine-1-phosphate in an Src-dependent manner and is involved in the migration of tumor and endothelial cells [46] (described in Section 2.5.2). This phosphorylation is important for MT1-MMP association with an adaptor protein p130cas (CRK-associated substrate) [49]. p130cas is an important substrate of Src and is dominantly localized in FAs of adherent cells, and plays a central role in integrin-mediated control of cell behavior [128]. Moreover, MT1-MMP is targeted to FAs through an interaction with a FAK (focal adhesion kinase)–p130cas complex [129]. Disruption of these interactions results in a significant reduction in ECM degradation at FAs but not at invadopodia, suggesting an FA-specific, Src-regulated mechanism. Further analysis revealed that the FAK–MT1-MMP interaction is mediated by the PRR domain of FAK and the CT of MT1-MMP. Direct interaction between FAK and MT1-MMP was, however, not detected and is believed to be mediated by p130cas [129].

The other phosphorylation site within the CT, Thr^567^ (see Section 2.5.1), was also shown to have a role in adhesion. Phospho-mimetic mutant T567E of MT1-MMP led to increased adhesion of ovarian cancer cells and multicellular aggregates to peritoneal explants relative to cells expressing wild-type or phosphodeficient mutant T567A [130]. It was reported that Thr^567^ is phosphorylated by integrin-linked kinase (ILK) [131]. ILK is a multifunctional protein that binds cytoplasmic domains of β-integrin and forms a ternary complex with PINCH (particularly interesting new cysteine-histidine-rich protein), and parvin termed IPP. This complex has a role in the adhesion and organization of the actin cytoskeleton downstream of integrins [132]. However, it is questionable whether Thr^567^ of MT1-MMP can actually be phosphorylated by ILK. It was shown that ILK is a pseudokinase whose putative kinase activity is non-existent and, therefore, cannot be the means of enacting its function in vivo. Instead, the kinase homology domain is a critical mediator of several protein–protein interactions [132,133,134]. It is more likely that Thr^567^ phosphorylation is modulated during adhesion downstream of β1-integrin via PKC instead of ILK, as described in Section 2.5.1. How this phosphorylation contributes to increased adhesion is, at the moment, unclear.

## 7. Regulation of Invasiveness through the CT

Invasion—directed movement into surrounding tissue—is a mechanism employed by cells in both physiological (e.g., angiogenesis, tissue remodeling, development) and pathological (e.g., cancer metastasis) contexts. Various invasion modes can be employed by invading cells that differ in their requirement of proteolytic activity, thus showing varying dependency on MT1-MMP. Amoeboid cells push through pores within the ECM by dynamical propulsions enabled by actomyosin contractility and membrane blebbing. This type of migration is preferably adopted in extracellular environments that allow for cell passage without the necessity to digest the ECM. On the other hand, collective and mesenchymal migration depends on proteolytical degradation of the ECM in the proximity of the cell body to create tunnel-like passages large enough for direct translocation of the cell body. This also enables invasion in dense matrices with constricting pore sizes [135,136,137].

MT1-MMP is one of the main enactors of mesenchymal invasion, as it depends on matrix degradation to make way for the cell [13]. On top of that, MT1-MMP also drives cell motility, which in itself is proteolysis-independent [102,138].

Promoting ECM rearrangement is the main role of MT1-MMP. Therefore most of the discussed mechanisms of regulation of MT1-MMP, such as post-translational modifications and trafficking, impact some aspects of the invasive capabilities of the cells that express it. Since the CT orchestrates many of these mechanisms, its deletion has been observed to lead to the loss of the MT1-MMP-induced proteolysis-independent promotion of migration [35,59,66,102,111], gelatine degradation [26,139], invasion into the matrix (while proteolytic activity stayed unaffected) [59], and even a significant decrease in capacity for tumor development in xenograft models [66].

However, other reports found the CT to be dispensable for migration [26], the proteolytic activity of the cells [59,140,141,142], MMP2 activation [66,141,143], and invasive capacity [140,142]. In some cases, the retention of the CT deletion mutant on the surface of the cells even led to an increase in proteolysis and invasion [26,144].

Looking more closely at the CT, several amino acids and motifs have been shown to influence cell invasion. Phosphorylation of both Thr^567^ and Tyr^573^, discussed in earlier chapters, leads to a higher invasion rate. Cells carrying the phosphodeficient Thr^567^ mutant migrate and invade less than the WT despite the mutant being more abundant on the cell surface, and conversely, a phosphomimetic mutant or a high phosphorylation state increases migration and invasion [37,40,130]. Phosphorylation of Tyr^573,^ on the other hand, promotes migration, matrix degradation, and invasion while increasing MT1-MMP concentration on the surface [46,47,48]. It is also necessary for proliferation in 3D matrix gels [47,145].

Another regulator of MT1-MMP activity is MTCBP-1 (MT1-MMP cytoplasmic tail binding protein 1), which binds the PRR^570^ motif and inhibits MT1-MMP-mediated migration, ECM degradation, and invasion [146,147]. It prevents MT1-MMP from binding actin, an interaction that happens through the LLY^573^ motif directly adjacent to the binding site of MTCBP-1 and is mediated by N-WASP (neural Wiskott-Aldrich syndrome protein) [139,147]. Disruption of the interaction with actin leads to the loss of targeting of MT1-MMP to invadopodia [147]. Overexpression of MTCBP-1 also reduces the number of invadopodia, possibly preventing the formation of new invadopodia [147]. It is of note that invadopodia are not only centers of ECM degradation but also adhesive structures, and the uncoupling of MT1-MMP from the actin cytoskeleton may affect adhesion as well. However, the role of MTCBP-1 in FAs and other MT1-MMP-regulated adhesive scenarios is yet to be tested.

The LLY^573^ motif is indispensable for invadopodia formation and invasion through ECM, likely due to it being the site of actin binding, the role of the motif on endocytosis of MT1-MMP, and the regulatory effect of the final tyrosine [35,59,139]. Interestingly, the dileucine motif itself is necessary for invadopodia formation [148]. LL^572^ regulates MT1-MMP glycosylation (Section 2.1) [18], which was shown to influence MT1-MMP function. Unglycosylated mutants were unable to process proMMP2, while collagenolysis and autoprocessing remained unaffected [17].

Cys^574^, which directly follows the LLY motif, is palmitoylated and plays a role in MT1-MMP endocytosis and homodimerization. It is not important for MMP2 activation, but it is crucial for proper adhesion, migration, and invasion [52,76].

Lastly, the C-terminal DKV^582^ motif, a binding site for PDZ domain-containing proteins, is dispensable for MT1-MMP-mediated stimulation of motility [35], but it is necessary for the maturation and proteolytic function of invadopodia, and therefore invasion in ECM [76,96]. The ubiquitination of the lysine in this motif (Section 2.6) is also essential for invasion through type I collagen [51].

As experimental findings vary, it is difficult to make generalizing conclusions. For example, if we consider the fact that MT1-MMP needs the CT to be endocytosed initially, that might result in an increase in MT1-MMP activity on the surface as it accumulates there. However, in the long run, this might become disadvantageous as focal recruitment becomes impossible, and TIMP2 cannot dissociate since MT1-MMP does not pass through the late endocytic compartment [61,81]. Similarly, the level of expression contributes to the resulting phenotype of the mutant-containing cells [26]. Additionally, as described in Section 5, the CT plays a role in regulating major signaling pathways that receive multiple inputs and have a widespread effect on the behavior of the cell. Therefore the entire context the cell finds itself in majorly contributes to its resulting ability, or decision, to invade.

## 8. Conclusions

The cytoplasmic tail of MT1-MMP can affect many of the functions that we know MT1-MMP has in the cell, although most of these are carried out by its much larger extracellular part. The 20 amino acids that comprise the cytoplasmic tail of MT1-MMP are the only site of direct contact with the interior of the cell for this 582 amino acids long enzyme. Therefore it is unsurprising that there are many proteins that interact with this sequence in some way (Figure 1). Of particular note is the central region, where several interactors compete for binding (MTCBP-1, radixin, actin, AP-2, GRASP55) and which contains three sites of post-translational modifications (phosphorylation at Thr^567^ and Tyr^573^ and palmitoylation of Cys^574^). It should also be considered that it is likely that other proteins, whose interactions have not been described, bind this sequence. Additionally, there is a substantial number of indirect interactors, which crowd the limited space around the CT. The competition over a small sequence of the CT by several adhesion and migration-associated molecules suggests that the CT is indeed an important regulatory hub in the processes of cellular adhesion and migration. The CT serves to modulate the proteolytic function of MT1-MMP itself, as well as other pathways. MT1-MMP uses to contribute to the stimulation of the invasive and metastatic program.

It is currently unclear which interactions are mutually exclusive, apart from MTCBP-1 binding disrupting the interaction with actin [147]. Similarly, we do not have much information on how the post-translational modifications affect the binding of interactors, except the necessity for Cys^574^ palmitoylation for AP-2 binding [35]. It seems that Tyr^573^ and the final DKV^582^ are functionally related, seeing as LIMK1/2 binds the C-terminal motif and phosphorylates the tyrosine and, conversely, phosphorylation of Tyr^573^ is required for the ubiquitination of Lys^582^ [48,51]. These examples imply an interconnectedness and mutual influence between the interactors and modifications.

Given the involvement of MT1-MMP in regulating several important pathways of tumorigenesis and metastasis (Section 5, Section 6 and Section 7), developing inhibitors against the CT might not only hinder the invasion of cancer cells but also deregulate other aspects, such as the metabolism or adhesive properties of the cells.

## Data Availability

Not applicable.
